# Purification and characterization of a novel type of neurotoxic peptides from the venom of the Iranian scorpion *Hemiscorpius lepturus*

**DOI:** 10.22038/IJBMS.2019.37910.9025

**Published:** 2020-02

**Authors:** Mahboob Maleki, Naser Mohammadpour Dounighi

**Affiliations:** 1 Department of Biochemistry, Fars Science and Research Branch, Islamic Azad University, Fars, Iran; 2 Department of Biochemistry, Shiraz Branch, Islamic Azad University, Shiraz, Iran; 3 Department of Human Vaccine and Serum, Razi Vaccine and Serum Research Institute, Agricultural Research, Education and Extension Organization, Karaj, Iran

**Keywords:** Hemiscorpius lepturus, Purification, Scorpion venom, Toxic peptides, Toxin

## Abstract

**Objective(s):**

Scorpion venom has toxic effects on mammals, insects and crustaceans. Toxicogenic peptides are major contributors to the scorpion venom, which make it toxic. The *Hemiscorpius lepturus (H. lepturus)* is one of the most common scorpion bites agent, and responsible for 95% of scorpion bite deaths cases in Iran.

**Materials and Methods:**

In this project, we fractionated the *H. lepturus* scorpion venom and analyzed toxic fractions of the venom. The crude venom of *H. lepturus* was dialyzed against distilled water and then the soluble part of the venom was isolated from the non-soluble (mucoproteins) part of the venom and loaded onto the Sephadex G-50 gel filtration column, then after determining the toxicity of the obtained fractions (fractions toxicity were detected in mice by IV injection), the resulting toxic fraction was purified with three stages of ion-exchange chromatography (anion and cationic) and RP-HPLC. The purity of the fractions was verified by SDS-PAGE electrophoreses.

**Results:**

The LD_50_ of *H. lepturus* venom was 177.01 µg/mouse. The crude venom had 7 detectable bands with molecular weights of 10-100 KDa and one band less than 10 KDa. Finally, after the different stages of chromatography, two HL2153 and HL2155 peaks were obtained from the RP-HPLC, which were depicted single bands and high purity. The electrophoretic analysis showed molecular weight 4874 Da for HL2153 and 5107 Da for HL2155 toxins.

**Conclusion:**

It is concluded that *H. lepturus* venom contains two HL2153 and HL2155 toxins with a relatively similar molecular weight and similar electrical charge 4874 and 5107 Da, respectively.

## Introduction

*Hemiscorpius lepturus* (*H. lepturus*) is the most dangerous scorpion of Khuzestan, the south-west, hot and humid province of Iran. A clinical study from Khuzestan province showed that *H. lepturus* was responsible for 12% of reported stings but it responsible for 95% of mortalities (1), that is probably due to the various pathological enzymes in its venom like hemitoxin, hemicalcin and heminecrolysin. The usual clinical manifestations of envenomation by *H. lepturus* include dermo-necrotic reactions, anemia, hemolysis, renal failure, and cardiovascular and central nervous system disorders (2, 3).

As written information on Chinese herb states, snake and scorpion venoms contain many different biologically active proteins and peptides that have pharmacological activities such as antimicrobial, anti-epileptic, and channel-blocking activities (4-7).

The toxic fractions are classified according to structural and physiological effects into several families and subfamilies of distinct peptides. These peptides are classified based on their molecular size and activity towards membrane bound receptors, known as ion channels. They can cause an abnormal depolarization of excitable and non-excitable cells (8); which, if not treated on time can be fatal (9). The first group is formed by long chain peptides containing 60–76 amino acids residues which mainly affect sodium channels (10). The second group is represented by short chain peptides containing 21–40 amino acids residues which are active on potassium, chloride and calcium channels (10-13). In China, scorpions have been used in treatment of convulsion and epilepsy by Chinese traditional doctors since the Sung Dynasty (A.D. 960-1279). The venom of this species is used in the production of scorpion polyvalent antiserum (14).

Anticancer therapy is one of the major applications for the using of venom proteins and peptides. Some isolated proteins or peptides specifically bind to the cancer cell membrane and affect the cancer cell migration and proliferation (15). Research for new antimicrobials or antibacterial prototypes is continuously necessary for drug design and development seeking the treatment of infections involving multidrug-resistant microorganisms. It is also of considerable interest to explore and develop antimicrobials with a new mechanism(s) of action which can potentially avoid the appearance of drug resistance (16).

Because of their evolutionary time, their medical importance and the presence in their venomous glands of a variety of biologically active component, scorpions are used in an enormous variety of approaches and interdisciplinar studies (17). It has been estimated that 100.000 distinct peptides exist in scorpion venoms, but only limited number of these peptides have been described (18, 19). 

The aim of the present work was isolation and identification new toxic peptides from the Iranian scorpion *H. lepturus*, this were carried out by a hybrid chromatographic procedure consisted of gel filtration, ion-exchange and reverse-phase-high performance liquid chromatography (RP-HPLC). 

## Materials and Methods

***Chemicals***

The *H. lepturus* venom was supplied by the Razi Vaccine and Serum Research Institute (Karaj, Iran) as a lyophilized powder. The dialysis bag (cutoff ≤1200 Da), Sephadex G50, DEAE-Sepharose Fast Flow, CM-Sepharose CL-6B, and protein Mw marker were purchased from Sigma-Aldrich Co (St Louis CO, USA). The ammonium acetate, polyethylene glycol (PEG 6000), tris base, sodium acetate, acetonitrile, trifluoroacetic acid, NaCl, sodium dodecyl sulfate (SDS) solution (10 %w/v), acrylamide (bis-acrylamide 30 %w/v), and N,N,N’,N’-tetramethyl ethylenediamine (Temed) were purchased from Merck (Germany). All other materials were analytical grade. 

***Experimental***

*Protein content analysis*

The protein content of *H. lepturus *venom and venom fractions in different steps of study was measured by Bradford’s spectroscopic (UNICAM UV/Vis Spectrometer) method (20) by using bovine serum albumin as a standard.

*Toxicity*

The *in vivo* toxic activity of crude venom and fractions of venom was measured by intravenous (IV) injection in mice (albino mice, 18-20 g) using Spearman-karber method (21). In brief, for this test, mice were divided into 6 groups, include 4 mice in any group. Various quantities of venom was administered into mice in each group. The numbers of death of mice in each group was observed after 24 hr and the lethality value (LD_50_) of venom determined.

*Venom solution preparation*

The lyophilized powder of venom (500 mg) was dissolved in 30 ml cold double-distilled water, dialyzed overnight against cold double-distilled water in cold room (4 ^°^C), and centrifuged at 15,000 g, 20 min (Sigma-6k15) to precipitate the non-soluble materials. The supernatant was clarified using 0.45 µm and 0.2 µm filters and stored at -20 ^°^C until use (22-24).

*Gel filtration of H. lepturus venom*

The venom solution was subjected to a Sephadex G50 column (size 150×2.3 cm), size exclusion chromatography using 0.1M ammonium acetate (pH 6.8) mobile phase, flow rate 48 ml/hr as previously described (17). The eluted solution fractions (10 ml/tube) were collected in 100 tubes using a fraction collector (Pharmacia LKB FRAC100). The absorbance of fractions was determined at 280 nm using a Spectrometer (UNICAM UV/Vis Spectrometer), and the absorption curve of these fractions was plotted against their numbers. In this study, content of the fractions related to each peaks were mixed together and concentrated by dialysis against PEG for 24 hr, and kept frozen at −20 ^°^C until use (25). In order to determine the toxicity of peak solutions, samples were injected (IV) into mice as previously described (21). The toxic peak solutions further purified by ion-exchange chromatography.

***Ion-exchange chromatography***

*Anion-exchange chromatography*

The solution of peak 2 obtained from Sephadex G50 column (HL2) were loaded on DEAE-Sepharose column (size 50×1.6 cm), pre-equilibrated with 20 mM tris base pH 8.3 buffer, and the proteins were eluted at a flow rate of 30 ml/hr with a linear gradient of 300 ml of 20 mM tris base (pH 8.3) and 300 ml of the same buffer containing 0.5 M NaCl. The eluted fractions (5 ml) were collected using a fraction collector (Pharmacia LKB FRAC100). Absorbance was measured at 280 nm using a Spectrometer (UNICAM UV/Vis Spectrometer), and the absorption curve of these fractions was plotted against their numbers. The content of the fractions related to each peaks were mixed together and freeze dried and kept at -20 ^°^C (26). All of these peak solutions sampled for *in vivo* toxicity test, samples were injected IV into mice as previously described (21). The toxic peak solutions further purified by cation-exchange chromatography.

*Cation-exchange chromatography*

The toxic fraction solution (HL21) was submitted to cation-exchange chromatography on CM-Sepharose. The CM-Sepharose column (size 20×1.6 cm) were previously equilibrated with 20 mM sodium acetate buffer (pH 4.8). The proteins were eluted at a flow rate of 30 ml/hr with a linear gradient of 250 ml of 20 mM sodium acetate and 250 ml of the same buffer containing 0.5 M NaCl. The eluted solution fractions (5 ml) were collected using a fraction collector (Pharmacia LKB FRAC100). The absorbance of fraction solutions was determined at 280 nm using a Spectrometer (UNICAM UV/Vis Spectrometer), and the absorption curve of these fractions was plotted against their numbers. The content of the fractions related to each peaks were mixed together and freeze dried and kept at -20 ^°^C (26, 27). In order to determine the toxicity of peak solutions, samples were injected IV into mice as previously described (21). The toxic peak solutions further purified by RP-HPLC.

*RP-HPLC*

RP-HPLC was performed using HPLC system (Amersham pharmacia AKTA Explorer-HPLC,P-900) at a flow rate of 0.5 ml/min for 60 min. The fractions were separated by C8 column (250 mm×4.6 mm, 5 μm particle size, 100Ǻ pore size) with a linear gradient of solution A (0.1 % trifluoroacetic acid in water) and solution B (0.1% TFA in acetonitrile), 0-15% for 15 min, followed by 15-100% over 25 min, 100% over 5 min, and 100-0% over 5 min. The absorbance of protein solution fractions were detected at 215 and 280 nm, and collected manually, freeze dried and stored at -20 ^°^C (28). 

*SDS -polyacrylamide gel electrophoresis (SDS-PAGE)*

SDS-PAGE analysis (Mini-PROTEAN Tetra Cell, BIO RAD) was carried out on polyacrylamide gel (20 %w/v) containing 1 %w/v SDS in Tris–glycine buffer as described by (29). The protein bands in the polyacrylamide gels were stained with silver nitrate (30). 

## Results

***Crude H. lepturus venom characteristics***

The crude venom of *H. lepturus *used in this work depicted mouse LD_50_ 177.01 µg in toxicity test. The supernatant obtained from crud venom clarification showed protein content 123 mg, approximately 21.95 %w/w of the crude venom (Table 1). 

***Size exclusion chromatography***

**Figure 1:**
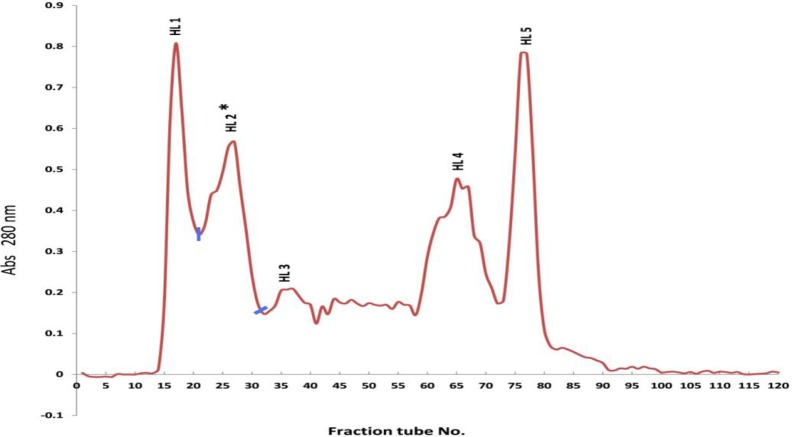
Gel filtration chromatogram of *Hemiscorpius lepturus* crude venom: The crude venom amount 500 mg, stationary phase Sephadex G50, column 150×2.3 cm, mobile phase ammonium acetate buffer, absorbance wave number 280 nm, and collection tubes 10 ml/tube

As shown in Figure 1, totally 5 distinct peaks were obtained (HL1, HL2, HL3, HL4, and HL5) pooled as indicated. The solution of peak 2, which named as HL2, shown toxic activity. 

***Anion-exchange chromatography***

**Figure 2:**
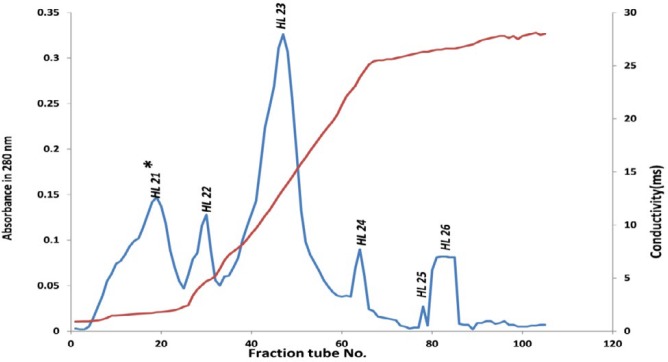
Anion-exchange chromatogram of HL2 peak obtained from gel filtration: stationary phase DEAE-Sepharose, column 50×1.6 cm, elution flow rate with a linear gradient of 300 ml of 20 mM tris base (pH 8.3) and 300 ml of the same buffer containing 0.5 M NaCl, and absorbance wave number 280 nm, and collection tubes 5 ml/tube

The peak 2 (HL2) from size exclusion chromatography was then further purified with DEAE-Sepharose column. As shown in Figure 2, totally six protein peaks were obtained from anion-exchange chromatography (HL21, HL22, HL23, HL24, HL25, and HL26) and the toxic fraction was found to locate in peak 1, which named as HL21. 

***Cation-exchange chromatography***

**Figure 3.**
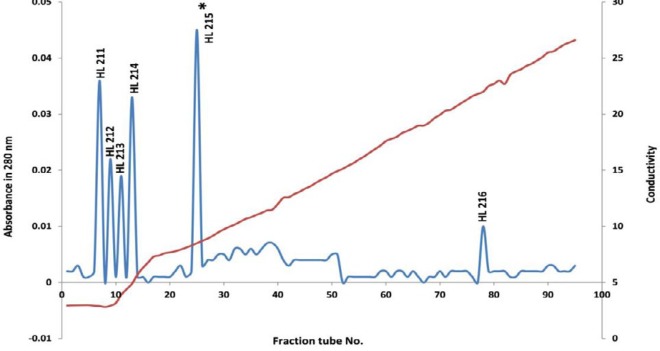
Cation-exchange chromatogram of HL21 peak obtained from anion-exchange chromatography: stationary phase CM- Sepharose, column 20×1.6 cm, elution flow rate 30ml/hr with a linear gradient of 250 ml of 20 mM sodium acetate and 250 ml of the same buffer containing 0.5 M NaCl, and absorbance wave number 280 nm, and collection tubes 5 ml/tube

The peak 1 (HL21) from anion-exchange chromatography was then further purified with CM-Sepharose column. As shown in Figure 3, totally six protein peaks (HL211, HL212, HL213, HL214, HL215, and HL216 were obtained from cation-exchange chromatography, and the toxic fraction was found to locate in peak 5, which named as HL215. 

***RP-HPLC***

**Figure 4:**
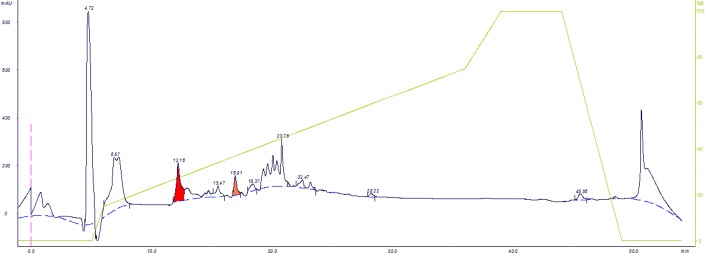
RP-HPLC chromatogram of HL215 obtained from Cation-exchange chromatography: C8 column with a linear gradient of solution A (0.1% trifluoroacetic acid in water) and solution B (0.1% TFA in acetonitrile). The protein fractions were detected at 215 and 280 nm. Totally 10 protein peaks were obtained and pooled

To obtain the ultra-pure sample, peak 5 (HL215) was pooled and further purified with reverse-phase C8 column. Ten protein peak were obtained (Figure 4), the toxic fractions were found to locate in peaks 3 and 5, which named as HL2153 and HL2155, respectively. 

***SDS-page electrophoresis***

**Figure 5:**
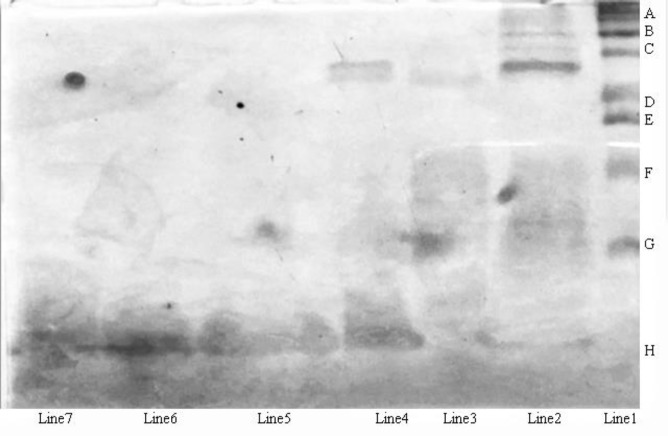
SDS-PAGE of crude *Hemiscorpius lepturus* venom and toxic protein peaks from chromatographic columns: Crude venom (Line 2), HL2 (Line 3) from Sephadex G50 column, HL21 (Line 4) from DEAE-Sepharose column, HL215 (Line 5) from CM- Sepharose column, HL2153 (Line 6) and HL2155 (Line 7) from RF-HPLC system were run on 20% SDS-PAGE under non-reducing condition. Line 1 shows the protein molecular weight marker: (A) 100 kDa, (B) 75 kDa, (C) 50 kDa, (D) 37 kDa, (E) 25 kDa (F) 20 kDa,(G) 15 kDa, (H) 10 kDa

The protein components of crude *H. lepturus* venom and each toxic peaks were analyzed by SDS-PAGE electrophoresis (Figure 5). The peaks of HL2153 and HL2155 depicted single bands and high purity. The apparent molecular weight of HL2153 and HL2155 were estimated respectively as 4.874 and 5.107 KDa by SDS-PAGE. 

## Discussion

Determination of biochemical properties of scorpion venom fractions and their mode of actions has been significant interest in recent years. A large number of toxins have been isolated, purified and characterized from various scorpion species (17, 18, 31-36). The toxic action of the scorpion venom is probably due to a some low molecular weight peptide toxins (36). The *H.lepturus* is one of the most important dangerous scorpion in Iran, which is distributed in southern cities, especially Khuzestan (37). The venom of this scorpion is a heterogeneous and pharmacologically important (4-7). 

This research was conducted to isolate toxic elements of *H.lepturus* scorpion venom by combining different chromatographic methods including gel filtration, ion-exchange chromatography, and RP- HPLC. Almost the most researchers have used the sephadex G-50 to initially isolate scorpion toxins (17, 38) but some used two-step HPLC to purify toxins of *Buthacus Macrocentrus* (39). 

The gel filtration of *H.lepturus* crude venom on Sephadex G-50 was revealed five protein pick (Figure 1), only fraction 2 (HL2) showed toxic activity (Table 2). In a similar study on *H.lepturus* venom fractionation by gel filtration chromatography on Sephadex G-50 four protein peaks were reported. In this study similar to our results the peak 2 was toxic (40). 

Toxic fraction HL2 obtained from gel filtration of *H. lepturus* venom further purified using DEAE-Sepharose anion-exchange column chromatography, which resulted in 6 protein fractions (Figure 2). In this study only the first fraction (HL21) was toxic (Table 3). In this work, for achievement high purity toxic protein fraction, the HL21 solution was fractionated by CM-sepharose cation-exchange chromatography. In this chromatography stage 6 protein peaks were revealed (Figure 3), only the fifth fraction HL215 was toxic (Table 4). The HL215 fraction was highly purified using RP-HPLC. Although the toxic fractions from the gel filtration can be directly injected in the HPLC column, but most of obtained fractions would be semi-purified. The protein fractions with similar amino acid sequences are closely interlinked and for the separation and purification of these proteins in addition to the analytical column, a preparative column is needed (41). When an extra chromatography step is performed before HPLC, many non-toxic substances are separated from toxic substances, which themselves results in a reduction in the number of RP-HPLC fractions, so it is possible to distinguish these fractions and make them recognizable (38, 41). Previously, The *Androctonus mauretanicus* scorpion venom (42) and *H. lepturus* scorpion venom (40) were fractionated by RP-HPLC system after gel filtration on Sephadex G-50, *H. lepturus* scorpion venom resulted in 25 fraction in RP-HPLC. In this work, we purified toxic fraction obtained from gel filtration using cation and anion exchange chromatography, before HPLC, which made it possible to obtain less and highly pure fractions by HPLC. Our results were shown 10 fractions in the HPLC chromatography for the HL215 fraction (Figure 4), that fractions of HL2153 and HL2155 obtained from HPLC were toxic (Table 4).

While, the previous report on RP-HPLC fractionation were revealed 25 fractions, only the 5^th^ fraction, called Hemicalcin, has been toxic (40). It is possible this difference reflects the high purity of the toxins derived from our work. To confirm the purity of the toxins obtained from the HPLC stage, electrophoresis and repeated HPLC have been used by most researchers (41, 43, 44). 

It seems that the toxins obtained from purification of the *H. lepturus* scorpion venom (HL2153 and HL2155) have a relatively similar molecular size and electrical charge, because they were presented in same fractions by gel filtration and ion-exchange chromatography. Miranda *et al.* believes that observing the single-band protein on the gel electrophoresis is essential to prove the fraction is homogeneous, but not sufficient, and confirming the final purity of the toxins and determining their molecular weight precisely is possible by analyzing amino acids sequence each toxin (25). Through analyzing amino acids, the probable molecular weight of toxins in the Buthidae family is reported to be 6,000-10,000 Da (25). In 2000, Hahin *et al.* reported the molecular weight of two toxins of BMK 9 (3) -1 and BMK 9 (3) -2 derived from *Buthus martensii karsch* scorpion venom 7020 and 7037 Da, respectively (45). 

**Table 1:** The amount of protein obtained from crude *Hemiscorpius lepturus* venom clarification

**Primary amount of crude venom (mg)**	**The amount of protein gained (mg)**	**Protein content of crude venom (%w/w)**
500	123	21.95

**Table 2:** Volume, protein content and percent yield of each peak obtained from the *Hemiscorpius lepturus* crude venom gel filtration

**Peak No**	**HL1**	**HL2**	**HL3**	**HL4**	**HL5**
Tube No.	13-20	21-31	33-43	58-72	73-81
Volume of peaks (ml)	80	110	110	150	90
Protein content of peaks (mg)	18.16	25.215	20.229	1.185	0.861
*percent yield of each peaks relative to crude venom protein (%w/w)	14.76	20.5	16.44	0.96	0.7

* The original crude venom protein was 123 mg

**Table 3:** Volume, protein content and percent yield of each peaks obtained from the HL2 anion-exchange chromatography

**Peak No.**	**HL21**	**HL22**	**HL23**	**HL24**	**HL25**	**HL26**
Tube No.	6-25	26-33	35-59	62-66	78	80-85
Volume of peaks (ml)	100	40	125	25	5	30
Protein content of peaks (mg)	5.39	0.49	5.86	0.535	0.095	0.593
*Percent yield of each peaks relative to the protein injected into the column (%w/w)	21.56	1.96	23.44	2.14	0.38	2.372

* The amount of protein injected into the column was 25 mg

**Table 4:** Volume, protein content and percent yield of each fraction obtained from the HL21 cation-exchange chromatography

**Peak No.**	**HL211**	**HL212**	**HL213**	**HL214**	**HL215**	**HL216**
Tube No.	7	9	11	13	25	78
Volume of peaks (ml)	5	5	5	5	5	5
Protein content of peaks (mg)	0.49	0.26	0.2	0.44	0.64	0.05
*Percent yield of peaks relative to the protein injected into the column (%w/w)	9.8	5.2	4	8.8	12.8	1

* The amount of protein injected into the column was 5 mg

**Table 5:** Protein content and yield percentage of RP-HPLC fractions for HL215 fraction

**Peak No.**	**HL2151**	**HL2152**	**HL2153**	**HL2154**	**HL2155**	**HL2156**	**HL2157**	**HL2158**	**HL2159**	**HL21510**
Protein content of peaks (g)	17.01	13.2	3.86	1.29	0.975	1.03	9.705	1.995	0.1	0.835
*Percent yield of peaks relative to the protein injected into the column (%w/w)	34.02	26.4	7.72	2.58	1.95	2.06	19.41	3.99	0.2	1.67

* The solution was injected into a column of 100 µl and a concentration of 0.05 mg/ml

## Conclusion

In this research, a simple and repeatable method for the isolation of toxins from venom of Iranian scorpion *H. lepturus* was presented. The results indicate that the two HL2153 and HL2155 toxins have a relatively similar molecular weight and similar electrical charge, because they were placed in a same fraction in the gel filtration and ion exchange chromatography. The approximate molecular weight of these toxins obtained by electrophoresis for HL2153 was approximately 4874 Dalton and HL2155 was approximately 5107 Dalton.
